# Increasing Rate of Fatal *Streptococcus pyogenes* Bacteriemia—A Challenge for Prompt Diagnosis and Appropriate Therapy in Real Praxis

**DOI:** 10.3390/microorganisms12050995

**Published:** 2024-05-15

**Authors:** Vaclava Adamkova, Vanda Gabriela Adamkova, Gabriela Kroneislova, Jan Zavora, Marie Kroneislova, Michal Huptych, Helena Lahoda Brodska

**Affiliations:** 1 Clinical Microbiology and ATB Centre, General University Hospital, 128 08 Prague, Czech Republic; 2 Department of Plant Sciences, University of Cambridge, Cambridge CB2 3EA, UK; 3 Department of Medical Microbiology, Palacky University, 779 00 Olomouc, Czech Republic; 4 Department of Clinical Pharmacy and Pharmacology, University Medical Center Groningen, University of Groningen, 9712 CP Groningen, The Netherlands; 5 Department of Surgery, University Hospital Bulovka, 180 00 Prague, Czech Republic; 6 Czech Institute of Informatics, Robotics and Cybernetics (CIIRC), Czech Technical University in Prague, 160 00 Prague, Czech Republic; 7 Clinical Biochemistry, General University Hospital, 128 08 Prague, Czech Republic

**Keywords:** *Streptococcus pyogenes*, GAS bacteriaemia, procalcitonin, lactate, myoglobin, appropriate therapy

## Abstract

*Streptococcus pyogenes*, group A streptococci (GAS) bacteriaemia, is a life-threatening infection with high mortality, requiring fast diagnosis together with the use of appropriate antibiotic therapy as soon as possible. Our study analysed data from 93 patients with GAS bacteraemia at the General University Hospital in Prague between January 2006 and March 2024. In the years 2016–2019 there was an increase in GAS bacteraemia. Mortality in the period 2006–2019 was 21.9%; in the period 2020–2024, the mortality increased to 41.4%, *p* = 0.08. At the same time, in the post-2020 period, the time from hospital admission to death was reduced from 9.5 days to 3 days. A significant predictor of worse outcome in this period was high levels of procalcitonin, >35.1 µg/L (100% sensitivity and 82.35% specificity), and lactate, >5 mmol/L (90.91% sensitivity and 91.67% specificity). Myoglobin was a significant predictor in both compared periods, the AUC was 0.771, *p* = 0.044, and the AUC was an even 0.889, *p* ≤ 0.001, respectively. All isolates of *S. pyogenes* were susceptible to penicillin, and resistance to clindamycin was 20.3% from 2006–2019 and 10.3% in 2020–2024. Appropriate therapy was initiated in 89.1%. and 96.6%, respectively. We hypothesise that the increase in mortality after 2020 might be due to a decrease in the immune status of the population.

## 1. Introduction

Sepsis, defined as life-threatening organ dysfunction caused by a dysregulated host response to infection, is a major cause of mortality from any infectious disease worldwide [1]. Epidemiological analyses increase general awareness and knowledge of infectious diseases, and molecular biological methods contribute to the identification of pathogenicity and virulence factors in causative agents, as well as to the elucidation of risk factors for the onset and development of infection in patients. Reducing mortality due to infections is a global public health priority [2]. In 2005, the WHO reported a global estimate of 18.1 million cases of severe *Streptococcus pyogenes* disease, with 1.78 million new cases of severe disease and 517,000 deaths per year [3].

*Streptococcus pyogenes*, group A streptococci (GAS), is a gram-positive, facultatively aerobic bacterium. The spectrum of infections caused by *S. pyogenes* is wide, ranging from respiratory tract infections, septic arthritis, puerperal sepsis and necrotizing fasciitis to streptococcal toxic shock syndrome. All these forms of the disease can be complicated by bacteraemia [4]. Streptococcal infections are highly contagious. Transmission most often happens from person to person, either by the droplet route or direct contact and, rarely, through contaminated food, leading to outbreaks of the disease. With few exceptions, streptococcal infections occur sporadically [5], although outbreaks of invasive infections caused by certain clones of *S. pyogenes* have been described, such as in Israel [6]. Most streptococcal infections are mild or self-healing [7]. However, in the last few years, there has been an increasing incidence of streptococcal infections in both children and adults worldwide, with an increase in invasive forms of infections [8,9,10,11,12,13].

The most common form of invasive GAS infection is bacteraemia (up to 75%). Localized infections without bacteraemia or necrotizing fasciitis occur less frequently—19% and 7%, respectively [9]. Although bacteraemia is the most common form of invasive infection among streptococcal infections, it is not one of the most common infections in comparison with other causative agents; its incidence does not exceed 8% [14,15]. However, among the causative agents causing bloodstream infections with the highest related mortality, *S. pyogenes* ranks fifth, behind *Escherichia coli, Klebsiella pneumoniae, Staphylococcus aureus* and *Pseudomonas aeruginosa* [14].

The pathogenesis of streptococcal infections is studied in detail, but the pathophysiology of mainly invasive forms is still unclear [16]. The essential condition for successful pathogenesis is the ability to invade and/or modulate the immune response. Patients with severe invasive GAS infections have been shown to have significantly lower serum vascular endothelial growth factor (VEGF) concentrations compared to those with non-invasive forms of infection [17]. *S. pyogenes* produces a variety of surface-bound, intracellularly produced and extracellularly produced factors, such as adhesins, pili, cytolysins, spreading factors and immune evasion factors that directly or indirectly influence the immune response. *S. pyogenes* also developed several strategies to evade the host immune response [18,19]. With the development of new diagnostic techniques, new immunomodulating enzymes have been identified, although not all of them have a clear role in the development of infection. Some of them could be used as biotechnological tools, or as drugs in the fight against autoimmune diseases, or as vaccine candidates [20,21,22]. Most enzymatic activities inhibit inflammatory processes, such as neutrophil chemotaxis mediated by IL-8 and C5a (via SpyCEP, ScpA), the inhibition of trapping and killing in neutrophil extracellular traps (via the DNAases SpnA and SdaI, as well as other streptococcal DNAses), the inhibition of killing by antimicrobial peptides (AMPs) and antimicrobial chemokines (both SpeB) and the killing of macrophages (via NADase, S5nA). Notable exceptions are SpeB’s activity on gasdermins that induce pyroptosis and SpeB’s activity on IL-1β and H-kininogen, which have potent proinflammatory effects [23]. *S. pyogenes* secretes a variety of highly mitogenic exotoxins that stimulate large numbers of T cells and antigen-presenting cells. This results in a massive release of pro-inflammatory cytokines and can lead to systemic inflammation and multiorgan failure [24].

The gold standard treatment for GAS sepsis is a combination of cell wall synthesis inhibitors (i.e., beta-lactams or glycopeptides) and protein synthesis inhibitors (MLS antibiotics) [25]. Although *S. pyogenes* is susceptible to penicillin in vitro [26], penicillin monotherapy treatment of GAS infections with toxin production has been associated with high morbidity and mortality due to the “Eagle effect” [27,28].

Worldwide increasing resistance of *S. pyogenes* to clindamycin might decrease its effect [29,30], with the surprising exception of Spain, where tetracycline, erythromycin and clindamycin resistance rates declined between 2007 and 2020 [31]. Data from the Czech Republic also do not show an increase in resistance to erythromycin and clindamycin [32]. The risk of resistance to beta-lactam antibiotics is very low due to *S. pyogenes*’ incapacity to transfer genes horizontally. Nevertheless, strains of *S. pyogenes* (emm43.4/PBP2x-T553K) with increased insensitivity to beta-lactams have already been described in the USA [33].

The use of biomarkers as possible predictors of aetiology in septic patients is repeatedly discussed. Nevertheless, currently there is no single commercially available biomarker that would be 100% specific and sensitive to discriminate between gram-negative and gram-positive sepsis [34]. This hinders clinical sepsis pathway implementation, potentially leading to an inappropriate choice of antibiotic therapy, which in the worst-case results in patient death [35].

Frequently used biomarkers, such as the total white blood cells, neutrophil count and C-reactive protein (CRP), lack the specificity to discriminate between SIRS and sepsis [34]. In this sense, procalcitonin (PCT)—a prohormone of calcitonin—was shown to have the best accuracy to identify patients with invasive bacterial infections because inflammatory stimuli including severe infection leads to its upregulated production in different tissues [36]. Even though elevated PCT serum concentrations are not exclusive to infections (they can also be elevated during paraneoplastic processes and in patients with solid tumours or with major trauma [37]), at this moment, PCT is considered among the best clinically available biomarkers to diagnose sepsis in routine praxis [38] and can be used as a guide to fulfil the principles of antimicrobial stewardship (AS). Using biomarkers to predict worse outcome in GAS sepsis has not been yet studied, to our knowledge.

In our retrospective study, biomarkers, routinely available in every healthcare facility, were evaluated and compared in patients with GAS bacteraemia in the period up to 2019, before COVID-19, and beyond. GAS sepsis with positive blood culture is rare [39], and, therefore, our cohort was unique due to its large number of GAS patients. The aim was to identify biomarkers with the highest informative value predicting a worse outcome of the disease or the risk of death of the patient.

## 2. Materials and Methods

### 2.1. Study Design and Setting, Inclusion Criteria

The present retrospective study included all patients admitted to the General University Hospital in Prague with *Streptococcus pyogenes* bacteraemia, confirmed through blood cultures, during the period between January 2006 and March 2024.

Only confirmed cases with complete data (PCT, CRP, neutrophil to leukocyte ratio (NLR), white blood cells count (WBC), blood cultures, albumin, lactate, creatinine, myoglobin, recommended antibiotic (ATB) treatment) were analysed. We obtained electronic medical health records for each patient from hospital information system.

The aim was to identify biomarkers with the highest informative value predicting a worse outcome and to evaluate the appropriateness of initial therapy.

### 2.2. Analysed Data

PCT, CRP, NLR, WBC, albumin, lactate, creatinine, myoglobin measurements were performed in hospital laboratory using commercially available assays as part of routine care. PCT was determined by chemiluminescence immunoassay (PCT normal values < 0.05 µg/L). CRP and myoglobin were determined by immunoturbidimetric method (CRP normal values < 0.5 mg/L, myoglobin 17–105 µmol/L Male(M) and 44–104 µmol/L Female (F)). Albumin was determined by photometric method (bromocresol green), (normal value: 35–53 g/L); creatinine was determined by photometric Jaffé method, normal value 44–110 µmol/L M, 44–104 µmol/L F. All analyses were determined by DXI Beckman Coulter; lactate was determined by ion selective electrode (ISE) methods by ABL 800 Flex, normal values 0.5–2.0 mmol/L. NLR and WBC were determined by flow cytometry (normal values < 3), Sysmex XE 5000.

All PCT values and other laboratory parameters were recorded within the first 24 h after the onset of sepsis as baseline data. Blood cultures were drawn at sepsis onset before the start of antimicrobial therapy and processed and analysed according to local laboratory standards. *S. pyogenes* was identified by latex agglutination. Antibiotic susceptibility was determined according to EUCAST rules. The investigators, considering all available clinical and microbiological data, identified the focus of infection retrospectively. For analysis, foci of infection were grouped into three categories (soft tissue, respiratory and other). Risk factors such as diabetes mellitus and chronic renal insufficiency were obtained from the patients’ medical records.

Appropriate treatment was defined as a combination of a beta-lactam antibiotic with an antibiotic that inhibits protein synthesis (macrolides, lincosamides, oxazolidinones); in the case of using only one appropriate antibiotic, the treatment was marked as semi-appropriate. Initial treatment with an antibiotic other than the ones mentioned above was evaluated as inappropriate.

### 2.3. Statistical Analysis

Continuous values were tested to normal distribution by the Shapiro–Wilk test, with negative results, and are expressed as medians and interquartile range (IQR). The categorical variables are expressed as a number and percentage (%). The continuous variables were compared by Kruskal–Wallis test for more than two groups and by 2-sided Mann–Whitney test for comparison of two groups (included post-hoc analysis). In the post-hoc analysis, the *p*-values were corrected by Bonferroni correction for multiple pairwise comparison. We performed 4 pairwise (clinically meaningful) comparisons; thus, we used Bonferroni correction by 4. The categorical values were compared using the Fisher’s exact test (for 2 × 2 table) or the chi-square test. The survival analysis was performed by Kaplan–Meier survival analysis. The Kaplan–Meier curves were compared by Logrank test. The ROC analysis and ROC comparisons (the hypothesis that the difference between the two ROC AUCs is 0) are utilized to emphasize the important differences between particular parameters. The *p* value lower than 0.05 was considered statistically significant. Statistical analyses were performed with MedCalc^®^ Statistical Software version 22.021 (MedCalc Software Ltd., Ostend, Belgium; https://www.medcalc.org; Accessed on 15 April 2024).

## 3. Results

### 3.1. Patient’s Population

**Figure 1:**
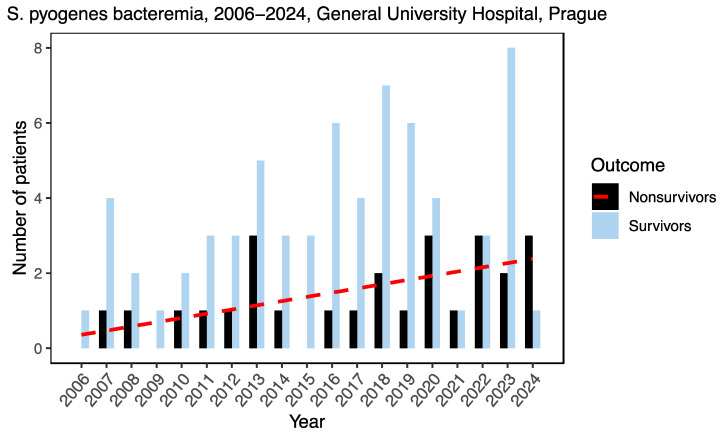
Incidence of *S. pyogenes* (GAS) bacteraemia in General University Hospital in Prague between January 2006 and March 2024. The red dotted line shows the increase in mortality rate represented by linear regressio.

During the 18-year-long study period, 93 patients with GAS sepsis were hospitalized in the General University Hospital, Prague, as shown in Figure 1. In the period 2006–2019, there were 64 patients, of which 36 were men (56.2%), and the median age in the group was 65.5 years (range 11–93 years, IQR (50.5–76)). For the period 2020–March 2024, 29 patients were recorded, of which 16 were men. The median age was 58 years (range 35–88, IQR (44.5–75.3)). During 2006–2019, 14 (21.9%) patients died, of which 5 (35.7%) were men; in the period 2020–2024, 12 (41.4%) died, of which 6 (50%) were men. The mortality almost doubled in the second follow-up period (*p* = 0.08). At the same time, the time interval from patient admission to death in non-survivors was significantly shortened, from 9.5 days (median, IQR 3–12) in the period 2006–2019 to 3 days (median, IQR 1–6.5), *p* = 0.02, in the follow-up period.

Diabetes mellitus and chronic renal insufficiency were recorded in both monitored periods, 14 (21.9%) patients in 2006–2019 and 4 (13.8%) patients in 2020–2024.

The majority of infections, i.e., 87 (93.5%), were community-acquired. The most common source of GAS bacteraemia in the monitored periods was infection of the skin and soft tissues (erysipelas, infected defect), 48 (75%) and 18 (62.1%), followed by respiratory tract infections, 6 (9.4%) and 6 (20.7%). Other sources of infection (CNS, digestive tract) were recorded in 10 (15.6%) and 5 (17.2%) patients in 2006–2019 and 2020–2024, respectively.

All strains of *S. pyogenes* in both study periods were susceptible to penicillin and linezolid. Resistance to macrolide antibiotics (erythromycin) and lincosamides (clindamycin) was 20.3% (13 strains) in the period 2006–2019, and, in 2020–2024, resistance was only 10.3% (3 strains).

Twelve (18.8%) patients received appropriate initial therapy in 2006–2019 and ten (34.5%) in 2020–2024, *p* = 0.12. Considering semi-appropriate therapies as appropriate, 57 (89.1%) patients met this criterion in the first follow-up period and 23 (79.3%) patients in the following period, *p* = 0.43. Even in the subpopulation of non-surviving patients, the inclusion of semi-appropriate therapies in the appropriate category did not have a significant impact on the evaluation of the cohort.

From the parameters of inflammation, no significant difference was found between the periods; the median C-reactive protein (CRP) was 235.1 mg/L (IQR: 113.6–349 mg/L) in the group up to 2019 and 252 mg/L (IQR: 128–312.8 mg/L), *p* = 0.97, in the group of patients from 2020–2024. A larger difference between the monitored periods was recorded for procalcitonin (PCT), namely 16.5 µg/L (median; IQR: 6.2–56.2 µg/L) and 37 µg/L (median; IQR: 5.9–68.4 µg/L), respectively, and *p* = 0.17. The median neutrophil–lymphocyte ratio was 22.5 (IQR: 14–31.6) and 27 (IQR: 9.8–37.8), respectively, and *p* = 0–79.

The severity of the condition is characterized by the lactate level, where the median in the period 2006–2019 was 2.7 mmol/L (IQR: 1.6–4.7 mmol/L) and, in the period 2020–2024, 5.0 mmol/L (IQR: 1.7–7.0 mmol/L), *p* = 0.12. In the case of myoglobin, the median was 895 µmol/L (IQR 250–7315 µmol/L) and 1294 µmol/L (IQR: 680–2783 µmol/L), respectively, *p* = 0.58.

The baseline value of creatinine and albumin in the first group was 139.5 µmol/L (median; IQR: 87.5–249.5) and 25 g/L (median; IQR: 20–28) or 147 µmol/L (median; IQR: 99–311.8) and 23.5 g/L (median; IQR: 21–31), respectively, and *p* = 0.48 and *p* = 0.77, respectively. All demographic, laboratory, and outcome clinical data are summarized in Table 1.

**Figure 2:**
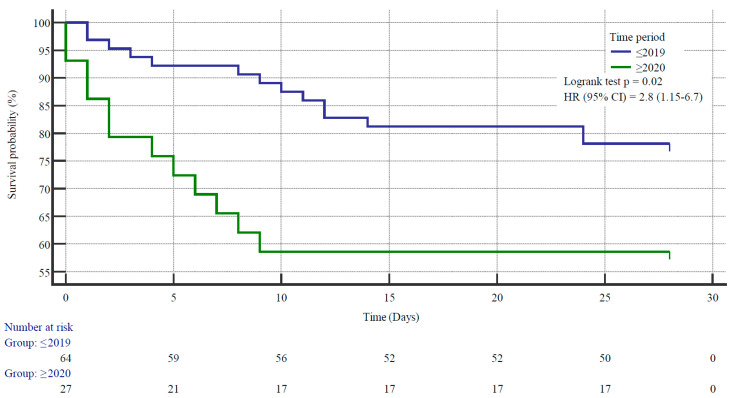
Kaplan–Meier survival curve of overall survival (in days) among patients with group A streptococci (GAS) bacteraemia hospitalised in General University Hospital, Prague, stratified by periods during which the patients were admitted to the hospital; 2006–2019 (in blue) and 2020–2024 (in green). The Kaplan–Meier curves were compared by Logrank test; *p* value < 0.05 was used as a cutoff.

Due to the significant change in the time from patient admission to death, Figure 2—the Kaplan–Meier survival curve, in the monitored periods—we decided to analyse individual parameters for the subgroups of patients in the periods 2006–2019 and 2020–March 2024. In the subgroup of non-surviving patients, there were 14 patients in the first period, of which 5 (35.7%) were men, and 12 patients in the following period, where the representation of men and women was equal, 6 and 6.

**Figure 3:**
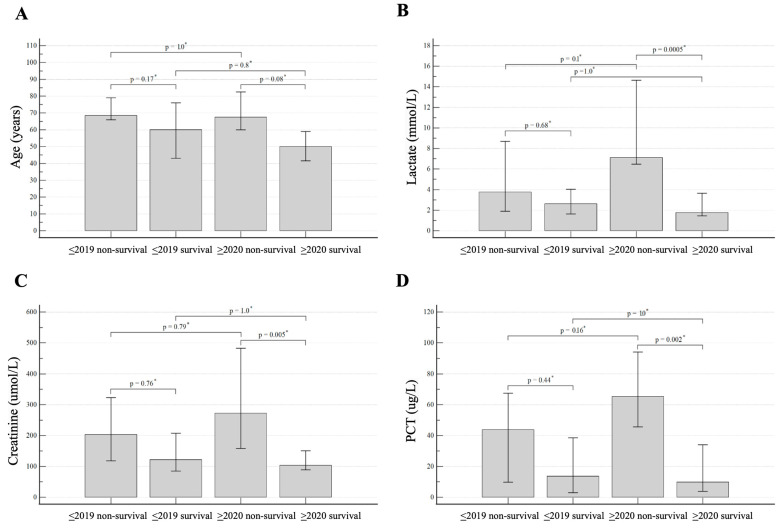
Post-hoc analysis of four pairwise comparison of survivors and non-survivors in periods 2006–2019 and 2020–2024 for clinically meaningful values: age (**A**), lactate (**B**), creatinine (**C**) and procalcitonin (PCT) (**D**). * The *p*-values are corrected by Bonferroni correction.

The comparison of individual groups of patients in the post-hoc analysis showed that the individual groups are comparable in age, with no statistical significance between survivors and non-survivors in both monitored periods (Figure 3A). There is a significant difference in the level of procalcitonin in surviving and non-surviving patients in the period 2020–2024—the median PCT is 9.47 μg/L and 67.5 μg/L, respectively, *p* = 0.002. As well, the prognostic significance of lactate is significant, with the median in survivors being 1.75 mmol/L and 7.1 mmol/L in non-survivors in the period 2020–2024, *p* = 0.005 (Figure 3B,D). An easily available marker in routine practice, creatinine, is also significantly elevated in non-surviving patients in the period 2020–2024, median 103 μmol/L vs. 272.5 μmol/L, *p* = 0.005) (Figure 3C).

### 3.2. Prognostic Performance of Biomarkers

**Figure 4:**
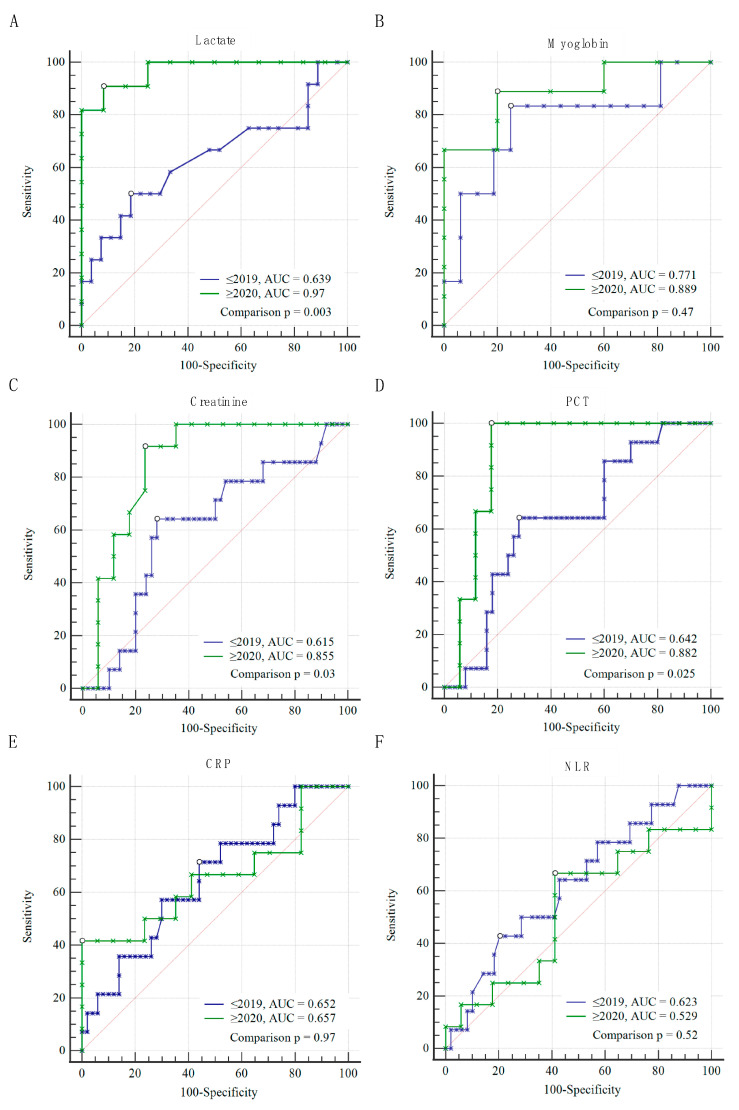
Comparison of receiver operating curve (ROC) analysis for lactate (**A**), myoglobin (**B**), procalcitonin (PCT) (**C**), creatinine (**D**), C-reactive protein CRP (**E**) and neutrophil–lymphocyte ratio (NLR) (**F**) to predict the worse outcome or the risk of death for patients admitted to General University Hospital in Prague between two periods, 2006–2019 (in blue) and 2020–2024 (in green). The pairwise comparison was used to compare the ROC between the two periods; *p* value < 0.05 was considered as significant. The * represents individual patients. The diagonal (red) line represents the theoretical ROC curve of random prediction. This diagonal line can be used as a reference for improving the data-based ROC curve versus random prediction.

When comparing routinely available biomarkers using receiver operation characteristic (ROC) curve analysis, high levels of lactate (AUC 0.97, *p* < 0.001) myoglobin (AUC 0.889, *p* < 0.001), PCT (AUC 0.882, *p* < 0.001) and creatinine (AUC 0.855, *p* < 0.001) were useful in predicting worse outcome or risk of death in patients with GAS bacteraemia in the patient cohort 2020–2024 only (Figure 4A–D). From these biomarkers, only myoglobin was useful to predict worse outcome in patients hospitalised before 2020 (AUC 0.771, *p* = 0.044) (Figure 4B). CRP and NLR could not be used to predict worse outcome in patients in either of the tested periods (Figure 4E,F). The best cut-off levels, according to the Youden index, for the 2020–2024 cohort were 5 mmol/L, with a sensitivity of 90.91% and specificity of 91.67% for lactate, 1039 µmol/L with a sensitivity of 88.89% and specificity of 80.00% for myoglobin, 35.1 µg/L with a sensitivity of 100% and specificity of 82.35% for PCT and 140 µmol/L with a sensitivity of 91.67% and specificity of 76.47% for creatinine. For the 2006–2019 cohort, the best cut-off value for myoglobin was 1073 µmol/L with a sensitivity of 83.33% and specificity of 75.00%. Sensitivity, specificity, cut-off values and Youden index J for individual biomarkers in the two tested periods are summarised in Table 2.

The incidence of diabetes mellitus was comparable in both groups (Table 1).

Appropriate antibiotic therapy was initiated in three (21.4%) non-surviving patients in the period 2006–2019 and in five (41.7%) in the following period, *p* = 0.4.

## 4. Discussion

This study presents a unique cohort of patients with GAS bacteraemia in one tertiary hospital between 2006 and 2024. We observed an increasing trend in the occurrence of GAS bacteraemia between 2016 and 2019. A similar trend was also reported in other studies worldwide [8,9,10,11,12,13]. Despite improving intensive care, source control and adequate antibiotic treatment, mortality in invasive GAS infections remains high, reaching up to 59% in the case of septic shock [40]. Mortality from 2016–2019 was 17.9%, lower than in subsequent years. Since 2019, mortality has increased, reaching 41.4% between 2020 and 2024. At the same time, the time from admission to the hospital to death has been reduced from a median of 9.5 days to 3 days. This fact is very alarming and was also a challenge for a more detailed analysis of the cohort of patients with GAS bacteraemia admitted to the hospital up to and from 2019, inclusive.

Blood culture (BC), the “gold standard” for the diagnosis of bloodstream infection, requires at least 48–72 h before the results of microorganism and antibiotic susceptibility are available. Due to the speed of development of symptoms of invasive GAS infection, traditional microbiological techniques for detection and identification of the infectious agent, based on cultivation methods, are slow and do not provide the necessary information in a timely manner [41]. The use of molecular biological methods, especially PCR, significantly shortens the time needed to clarify the etiological agent [42]. However, this type of diagnosis is not routinely available in all healthcare facilities in the Czech Republic and certainly not in the POCT regime.

Advanced life support and resuscitation together with prompt antibiotic treatment constitute a fundamental aspect of sepsis management. For adults with possible septic shock or a high likelihood for sepsis, administering antimicrobials immediately is recommended, ideally within 1 h of recognition of sepsis [43,44].

PCT and CRP are currently the most widely used inflammatory biomarkers in routine praxis in our country. PCT has shown a significant prognostic value even upon admission to the hospital, as lower serum levels have been associated with a higher probability of survival in patients with sepsis [45]. Upon admission, PCT levels are strongly related to the severity of the inflammatory reaction. PCT impairs the endothelial barrier function, causing capillary leak and refractory hypotension with subsequent multiple organ failure during sepsis [46].

In our study from 2020, we proposed the use biomarkers, especially PCT, in the differential diagnosis of GAS sepsis to improve the initial choice of antibiotics [47]. To our knowledge, we were the first to report that *S. pyogenes* can produce an inflammatory response similar to or higher than gram-negative (GNEG) bacteria [48]. *S. pyogenes*’ GNEG-like inflammatory response might be explained via recognition of the GAS pore-forming toxin streptolysin O by TLR4, leading to high expression of pro-inflammatory cytokines [49].

Common biomarkers, such as CRP, PCT, differential blood count, lactate or creatinine, are available everywhere in the Czech Republic, even in urgent mode. CRP has a high sensitivity for inflammation but little specificity for infection. CRP has a slower onset than PCT and peaks up to 24 h later. When interpreting CRP values, it is necessary to consider potential limitations (impairment of liver protein synthesis in severe hepatopathies and blocking of IL-6 during biological therapy—in both cases, CRP levels barely increase) [50].

CRP, as the most frequently determined marker, was elevated in both periods, median 235.1 mg/L and 252 mg/L; the difference was not significant: *p* = 0.97. Even when comparing the group of deceased patients in the observed analogues, the difference was not significant: 316.9 mg/L vs. 273 mg/L, *p* = 0.94. According to the Youden index, CRP determination could only be used as a screening test for worse outcome, where values higher than 326 mg/L have 100% specificity but only 41.7% sensitivity.

Although there was no significant difference in PCT levels in the overall comparison of either of the two periods, 16.5 µg/L vs. 37 µg/L, *p* = 0.17, the difference is still clinically relevant. However, when comparing surviving and non-surviving patients in the two tested periods, PCT values were significantly higher in non-surviving patients in the cohort 2020–2024. PCT has a high sensitivity and specificity for the detection of systemic infection. However, PCT levels increase not only at the instigation of PAMPs (pathogen-associated molecular pattens, e.g., liposaccharide in gram-negative), but also DAMPs (damage-associated molecular pattens, e.g., cell decay products). The advantage of PCT is its fast dynamics. PCT is used as a guide for antimicrobial therapy. Gram-negative infections are associated with higher levels of PCT than most gram-positive ones, except for pyogenic streptococci. PCT monitoring is not only diagnostic but also prognostic [51]. Our data show that unlike CRP, PCT can be used as a prognostic marker; values higher than 35.1 µg/L show 100% sensitivity and 82.35% specificity. The Youden index for this criterion is 0.8235.

The neutrophil–lymphocyte ratio can be used as a marker to differentiate between GAS sepsis and sepsis induced by other gram-positive bacteria [48]. However, in the group of patients with GAS bacteraemia, there are no differences between the observed periods; the median NLR was 22.5 vs. 27, *p* = 0–79. Similarly, even among non-surviving patients, the difference in the median NLR was not significant: 26.9 vs. 28.9, *p* = 0.63.

Lactate is a product of anaerobic glycolysis. It is used by gluconeogenesis as a source of energy. In systemic inflammation, the pathophysiology changes and lactate accumulates for various reasons (hypoxic-hypoperfusion type, hyperlactatemia in mitochondrial dysfunction, reduced pyruvate dehydrogenase complex activity, etc.). Sepsis-associated hyperlactatemia (SAHL) has been described [52]. The lactate level is a valuable marker to assess the severity of the condition, and monitoring lactate levels serves as a prognosis of patient’s condition [53]. In our cohort, although there was no significant difference between the study periods, medians of 2.7 mmol/L and 5.0 mmol/L, *p* = 0.12, there was a significant difference among the cohorts of deceased patients: 3.75 mmol/L vs. 7.1 mmol/L, *p* = 0.025. Similar to PCT, lactate can be used as a prognostic biomarker, where lactate levels higher than 5 mmol/L show 90.91% sensitivity and specificity of 91.67%; the Youden index for this criterion is 0.8258.

Similar to lactate, albumin levels also characterize the severity of the condition. Rapid changes in plasma albumin levels reflect the formation and the degree of capillary leakage in systemic inflammation. Therefore, a sharp drop in albumin levels can be used as a better estimate for the severity of the condition [54]. In our cohort, all patients had hypoalbuminemia on the day of admission; the difference between the cohort 2006–2019 and 2020–2024 was 25 g/L vs. 23.5 g/L, *p* = 0.77. When comparing deceased patients in the follow-up periods, the median albumin was 18.5 g/L in 2006–2019 and 24 g/L in the following period, *p* = 0.07. Recent studies pointed to the use of heparin-binding protein (HBP) as a potent indicator of worse outcome in patients with sepsis [55]. However, HBP is not routinely tested in the Czech Republic.

The most common source of bacteraemia was skin and soft tissue infections, found in 48 (75%) and 18 (62.1%) patients, respectively. The biomarker that reflects this is myoglobin. Indeed, myoglobin was the only biomarker that could predict worse outcome in patients in both study periods. In the 2006–2019 period, the AUC was 0.771 and the Youden index for criterion >1073 µmol/L was 0.5833, sensitivity 83.33%, specificity 75.00%, *p* = 0.044. In the period 2020–2024, the AUC was 0.889 and the Youden index for criterion >1039 µmol/L was 0.6889, sensitivity 88.89%, specificity 80.00%, *p* ≤ 0.001. Myoglobin is a muscle protein, and its elevated levels in the blood are found within two hours of muscle damage. Myoglobin has rapid dynamics, which is crucial for early patient monitoring. Furthermore, it serves to monitor the risk for renal damage, and, based on myoglobin levels, we decide when it is necessary to start haemodialysis [56,57]. On the other hand, creatinine phosphokinase has a slower release into the bloodstream, and there are no strict guidelines for monitoring of renal failure [57].

In contrast to the biomarkers mentioned above, creatinine is an indicator of kidney organ dysfunction. The kidneys are one of the first organs to respond to sepsis or septic shock. Sepsis-associated acute kidney injury (S-AKI) is a common complication in hospitalized and critically ill patients that increases the risk of developing chronic comorbidities and is associated with extremely high mortality [58]. As in other forms of AKI, serum creatinine can be an insensitive indicator of kidney injury. S-AKI is usually defined as AKI in the presence of sepsis without other significant contributing factors explaining AKI or characterized by the simultaneous presence of both Sepsis-3 and Kidney Disease: Improving Global Outcomes (KDIGO) criteria [59]. For patients in the ICU, sepsis is found in about 40% to 50% of patients with AKI [60]. A prospective cohort study including 1177 patients with sepsis across 198 ICUs in 24 European countries reported a 51% incidence of AKI with an ICU mortality rate of 41% [61]. In our cohort, the median baseline creatinine was 139.5 µmol/L in the period 2006–2019 and in the following period, 147 µmol/L, *p* = 0.48. Comparing deceased patients, the median creatinine was 203 µmol/L in the first period and 272.5 µmol/L in the second period, *p* = 0.2.

Analysis of biomarkers in a subpopulation of non-surviving patients between the monitored periods using ROC curves and determination of AUC showed that the only prognostic marker for the 2006–2019 period was myoglobin. However, in the period 2020–2024, we found that four biomarkers could be used to predict worse outcome or risk of death in patients, namely lactate, myoglobin, PCT and creatinine. This finding offers a new avenue to improve GAS sepsis management in the early hours of a patient’s admission to the hospital. The rate of development of a serious course of infection may also affect the suitability of initial antibiotic therapy.

The drug of choice for invasive infections caused by *S. pyogenes* remains the combination of a beta-lactam antibiotic with a protein synthesis inhibitor. The most commonly used protein synthesis inhibitor in the Czech Republic is clindamycin. A protein synthesis inhibitor with activity during the stationary phase of bacterial growth has been shown to decrease the expression and production of group A streptococcal virulence factors and exotoxins [62]. However, despite the unique anti-streptococcal properties of clindamycin shown in both in vitro and animal models [62], proof of its effectiveness in humans has been hampered by small sample sizes and low-quality clinical evidence. Observational studies that suggested benefits in patients with necrotising fasciitis [63], streptococcal toxic shock syndrome [64] and any invasive group A streptococcal infections [65] have not accounted for confounding effects by indication (i.e., the selective use of clindamycin [64]. In our file, 12 (18.8%) patients received full appropriate initial therapy in 2006–2019 and 10 (34.5%) patients in 2020–2024, *p* = 0.12.

Antibiotic monotherapy with an effect on *S. pyogenes* was evaluated as semi-appropriate in our study. Considering semi-appropriate therapies as appropriate, 57 (89.1%) patients met this criterion in the first follow-up period and 23 (79.3%) patients in the following period, *p* = 0.43. Given the significant reduction in the time from admission to death in the period 2020–2024, from a median of 9.5 days to 3 days, the question is whether appropriate therapy is entirely crucial. The rate of progression of the condition in these patients is striking. In one patient, a 64-year-old man, the course was so fulminant that he died early, within an hour of admission, before therapy could begin. The source of infection was an infected defect in the lower extremity. The baseline level of myoglobin was unmeasurable (>35,000 μmol/L), creatinine waS 495 μmol/L, procalcitonin was 102 μg/L and lactate was 11.2 mmol/L. The patient did not suffer from diabetes mellitus or chronic renal insufficiency and was not treated for malignancy as a risk factor for infection.

The lower adherence of the therapy in the second monitored period with higher mortality is not significant, nor can the addition of antibiotics with an effect on protein synthesis explain the higher mortality, because these antibiotics are bacteriostatic, and their onset of action is slower than that of beta-lactams [66].

More recently, concerns have arisen that clindamycin may no longer be the adjunctive antitoxin antibiotic of choice due to rising clindamycin resistance [67]. However, this trend is not uniform, and there is no increased resistance to clindamycin reported in the Czech Republic [32]. In our cohort, resistance to macrolide antibiotics (erythromycin) and lincosamides (clindamycin) was 20.3% (13 strains) in the period 2006–2019 and, in 2020–2024, resistance was only 10.3% (3 strains). So, the question remains why the mortality of our patients has increased so significantly since 2020.

The cause of the GAS resurgence observed in multiple countries has been widely debated [68,69]. It has been suggested that it might have resulted from non-pharmaceutical interventions that were implemented to limit SARS-CoV-2 transmission, but it also decreased the circulation of GAS. Hence, the population might have a reduced immunity to GAS [70,71]. Virulent GAS variants, established by the GAS M surface protein and encoded by the emm gene, have also been suggested to have influenced the high incidence of iGAS in 2022–2023 [72]. Since 2010, more invasive forms of GAS infections (such as scarlet fever) have been reported globally. In the UK, the resurgence of scarlet fever has been linked to a sub-lineage M1_UK_ of the pandemic M1T1 clone, which has an increased expression of SpeA superantigen [73]. In December 2022, there was an outbreak of GAS infection in London. Yet, the most prevalent M types were still *emm12* and *emm1.* The severity of GAS infection was associated with the presence of *spea* and *spej* superantigen genes [74]. In the Czech Republic, there was no change in *emm* types of *S. pyogenes* before and after the COVID-19 pandemic. *emm1* and *emm12* remain the most prevalent types in the Czech Republic (established via personal communication; data will be published). On the other hand, sequencing of GAS is not routinely performed, and, therefore, we cannot exclude the possibility that more virulent GAS is also present in the Czech Republic.

Reduced population immunity might have contributed to exceptionally high circulation of GAS and a proportional increase in invasive GAS (iGAS) infections [75]. *S. pyogenes* developed several strategies to evade the host immune response [18,19]. With the development of new diagnostic techniques, new immunomodulating enzymes have been identified, although not all of them have a clear role in the development of infection.

The possibilities of laboratory evidence of a reduced immune response to streptococcal infection are limited in the Czech Republic and completely unavailable in routine diagnostics. Given the high mortality rate caused by streptococcal infection, investigating the immune response in patients might need to be implemented in our hospital settings. One such marker is vascular endothelial grown factor (VEGF). VEGF play a role in the defence mechanism of GAS infection as it contributes to GAS clearance in endothelial cells. VEGF is an angiogenic factor involved in normal physiological functions, including bone formation, haematopoiesis, wound healing and development [76].

In endothelial cells, the invasive efficacy of GAS was shown to be 5-fold higher compared to epithelial cells, contributing to severe symptoms of GAS that were linked to the breakdown of blood vessels and dissemination of bacteria into the systemic circulation [77]. In endothelial cells, the autophagy and lysosomal functions are limited, which leads to a failure of suppression of bacterial proliferation [78]. VEGF was proposed as a key factor regulating the susceptibility of endothelial cells to GAS. Interestingly, the levels of VEGF are high at the sight of local infection, while, in patients exhibiting severe symptoms such as necrotizing fasciitis, sepsis and bacteraemia, the serum VEGF levels are low. In vitro, administration of VEGF increased the survival of GAS-infected mice. Furthermore, it was found that VEGF supresses GAS proliferation by enhancing lysosomal action and xenophagy in endothelial cells [17]. Given these findings, VEGF serum levels could potentially be used as a marker of disease severity as well as in the treatment of bacteraemia in the form of exogenous injections.

Due to the important role of VEGF in the angiogenesis of oncological diseases or eye diseases (especially macular degeneration of the retina) and the increase in use of VEGF inhibitors (e.g., Bevacizumab) in the treatment of these diseases, the question arises whether these patients, in addition to other undesirable drugs related to the administration of anti-VEGF (cardiotoxicity), will not be at increased risk of the onset and development of invasive GAS infections [79,80]. To understand the relationship between VEGF and GAS infection, further studies are needed.

One of the main limitations of our study is that it was retrospective and single-centre. Another limitation is the lack of data on M-protein typing of *S. pyogenes* (*emm* types), but due to the focus of the study on real practice, these data are not available because this is not routinely examined.

On the other hand, the cohort of 93 patients is unique in its characteristics of isolates obtained only from blood cultures. Based on the data obtained and the study of the patient records, we designed a unified algorithm for the laboratory examination of a patient admitted with suspected sepsis.

### Algorithm of Laboratory Examination of Patient with Suspected Sepsis

Within 1 h of the patient’s admission, a blood count with a differential count is determined, and Intensive Care Infection Score (ICIS) score, neutrophil–lymphocyte ratio (NLR), C-reactive protein, procalcitonin, lactate, iontogram, creatinine, urea, alanine aminotransferase (ALT), aspartate aminotransferase (AST), bilirubin, albumin and myoglobin are also determined in the urgent regimen. According to the results of the laboratory examination, together with the evaluation of the clinical condition, the most appropriate treatment is chosen.

## 5. Conclusions

In our retrospective study, we showed that there was a higher mortality rate in septic patients from 2020–2024. We demonstrated the importance in this period of procalcitonin and lactate as prognostic biomarkers of worse outcome or risk of patient death. Cutoff values of PCT > 35.1 μg/L and lactate > 5 mmol/L are indicative of a worse patient outcome. Furthermore, myoglobin was shown to predict worse outcome in patients in both tested periods. The result of our analysis is the design of a unified examination algorithm for patients with suspected sepsis in routine practice to initiate timely and appropriate antibiotic therapy. The combination of penicillin with a protein synthesis inhibitor remains the drug of choice in the Czech Republic. Based on these results, we are preparing a multicentre study to confirm the validity of our conclusions.

## Figures and Tables

**Table 1:** Demographics, clinical characteristics and outcomes of patients in the follow-up periods 2006–2019 and 2020–March 2024.

Parameter	≤2019	≥2020	*p*-Value
Gender, number (%) Male Female	36 (56.2) 28 (43.7)	16 (55.2) 13 (44.8)	1.0
Age, median years (IQR)	65.5 (50.5–76)	58 (44.5–75.3)	0.54
CRP (mg/L), median (IQR)	235.1 (113.6–349)	252 (128–312.8)	0.97
PCT (µg/L), median (IQR)	16.5 (6.2–56.2)	37 (5.9–68.4)	0.17
NLR, median (IQR)	22.5 (14–31.6)	27 (9.8–37.8)	0.79
Lactate (mmol/L), median (IQR)	2.7 (1.6–4.7)	5.0 (1.7–7.0)	0.12
Creatinine (µmol/L), median (IQR)	139.5 (87.5–249.5)	147 (99–311.8)	0.48
Myoglobin (µmol/L), median (IQR)	895 (250–7315)	1294 (680–2783)	0.58
Albumin (g/L), median (IQR)	25 (20–28)	23.5 (21–31)	0.77
Appropriate therapy Yes (only yes) No	12 (18.8) 52 (81.2)	10 (34.5) 19 (65.5)	0.12
Appropriate therapy Yes (yes and semi-) No	57 (89.1) 7 (10.9)	28 (96.6) 1 (3.4)	0.43
Diabetes Mellitus Yes No	14 (21.9) 50 (78.1)	4 (13.8) 25 (86.2)	0.41
Survival Yes No	50 (78.1) 14 (21.9)	17 (58.6) 12 (41.4)	0.08
Days to death, median (IQR) for non-survival only	9.5 (3–12)	3 (1–6.5)	0.02

Abbreviations: IQR, inter-quartile range; CRP, C-reactive protein; PCT, procalcitonin; NLR, neutrophil to leukocyte ratio.

**Table 2:** Accuracy and cut off values of individual biomarkers for predicting worse outcome or death of patients admitted to General University Hospital, Prague, in the follow-up periods 2006–2019 and 2020–March 2024.

Biomarker	Youden Index J	Cut-Off	Sensitivity	95% CI	Specificity	95% CI
CRP (2006–2019)	0.2743	>216	71.43	41.9–91.6	56.00	41.3–70.0
CRP (2020–2024)	0.4167	>326	41.67	15.2–72.3	100.00	80.5–100.0
PCT (2006–2019)	0.3629	>29.47	64.29	35.1–87.2	72.00	57.5–83.8
PCT (2020–2024)	0.8235	>35.1	100.00	73.5–100.0	82.35	56.6–96.2
NLR (2006–2019)	0.2245	>31.2	42.86	17.7–71.1	79.59	65.7–89.8
NLR (2020–2024)	0.2549	>26.4	66.67	34.9–90.1	58.82	32.9–81.6
Lactate (2006–2019)	0.3148	>4.4	50.00	21.1–78.9	81.48	61.9–93.7
Lactate (2020–2024)	0.8258	>5	90.91	58.7–99.8	91.67	61.5–99.8
Creatinine (2006–2019)	0.3629	>171	64.29	35.1–87.2	72.00	57.5–83.8
Creatinine (2020–2024)	0.6814	>145	91.67	61.5–99.8	76.47	50.1–93.2
Myoglobin (2006–2019)	0.5833	>1073	83.33	35.9–99.6	75.00	47.6–92.7
Myoglobin (2020–2024)	0.6889	>1039	88.89	51.8–99.7	80.00	28.4–99.5

Abbreviations: CRP, C-reactive protein; CI, confidence intervals; PCT, procalcitonin; NLR, neutrophil to leukocyte ratio.

## Data Availability

The original contributions presented in the study are included in the article. Further inquiries can be directed to the corresponding authors.

## References

[1] Rello J., van Engelen T.S.R., Alp E., Calandra T., Cattoir V., Kern W.V., Netea M.G., Nseir S., Opal S.M., van de Veerdonk F.L. (2018). Towards precision medicine in sepsis: A position paper from the European Society of Clinical Microbiology and Infectious Diseases. Clin. Microbiol. Infect..

[2] Gouel-Cheron A., Swihart B.J., Warner S., Mathew L., Strich J.R., Mancera A., Follmann D., Kadri S.S. (2022). Epidemiology of ICU-onset bloodstream infection: Prevalence, pathogens, and risk factors among 150,948 ICU patients at 85 U.S. hospitals*. Crit. Care Med..

[3] Carapetis J.R., Steer A.C., Mulholland E.K., Weber M. (2005). The global burden of group A streptococcal diseases. Lancet Infect. Dis..

[4] Barnett T.C., Bowen A.C., Carapetis J.R. (2018). The fall and rise of Group A *Streptococcus* diseases. Epidemiol. Infect..

[5] Efstratiou A., Lamagni T., Ferretti J.J., Stevens D.L., Fischetti V.A. (2016). Epidemiology of *Streptococcus pyogenes*. Streptococcus Pyogenes: Basic Biology to Clinical Manifestations.

[6] Ron M., Brosh-Nissimov T., Korenman Z., Treygerman O., Sagi O., Valinsky L., Rokney A. (2022). Invasive Multidrug-Resistant emm93.0 *Streptococcus pyogenes* Strain Harboring a Novel Genomic Island, Israel, 2017–2019. Emerg. Infect. Dis..

[7] Walker M.J., Barnett T.C., McArthur J.D., Cole J.N., Gillen C.M., Henningham A., Sriprakash K.S., Sanderson-Smith M.L., Nizet V. (2014). Disease manifestations and pathogenic mechanisms of Group A *Streptococcus*. Clin. Microbiol. Rev..

[8] Shakoor S., Khan E., Mir F., Malik F.R., Jamil B. (2017). Secular trends of *Streptococcus pyogenes* sepsis in Pakistan and analysis of clinical features in a hospitalized cohort. Trop. Biomed..

[9] Meehan M., Murchan S., Gavin P.J., Drew R.J., Cunney R. (2018). Epidemiology of an upsurge of invasive group A streptococcal infections in Ireland, 2012–2015. J. Inf. Secur..

[10] Blagden S., Watts V., Verlander N.Q., Pegorie M. (2020). Invasive group A streptococcal infections in North West England: Epidemiology, risk factors and fatal infection. Public Health.

[11] Vilhonen J., Vuopio J., Vahlberg T., Gröndahl-Yli-Hannuksela K., Rantakokko-Jalava K., Oksi J. (2020). Group A streptococcal bacteremias in Southwest Finland 2007–2018: Epidemiology and of infectious diseases consultation in antibiotic treatment selection. Eur. J. Clin. Microbiol. Infect. Dis..

[12] Bläckberg A., Svedevall S., Lundberg K., Nilson B., Kahn F., Rasmussen M. (2022). Time to blood culture positivity: An independent predictor of mortality in *Streptococcus Pyogenes* bacteremia. Open Forum Infect. Dis. Ther..

[13] Thomson T.N., Campbell P.T., Gibney K.B. (2022). The epidemiology of invasive group A streptococcal disease in Victoria, 2007–2017: An analysis of linked datasets. Aust. N. Z. J. Public Health.

[14] GBD 2019 Antimicrobial Resistance Collaborators (2022). Global mortality associated with 33 bacterial pathogens in 2019: A systematic analysis for the Global Burden of Disease Study 2019. Lancet.

[15] Tabah A., Buetti N., Staiquly Q., Ruckly S., Akova M., Aslan A.T., Leone M., Conway M.A., Bassetti M., Arvaniti K. (2023). Epidemiology and outcomes of hospital-acquired bloodstream infections in intensive care unit patients: The EUROBACT-2 international cohort study. Intensive Care Med..

[16] Reglinski M., Sriskandan S. (2014). The contribution of group A streptococcal virulence determinants to the pathogenesis of sepsis. Virulence.

[17] Lu S.-L., Omori H., Zhou Y., Lin Y.-S., Liu C.-C., Wu J.-J., Noda T. (2022). VEGF-mediated augmentation of Autophagic and lysosomal activity in endothelial cells defends against intracellular *Streptococcus pyogenes*. mBio.

[18] Okumura C.Y.M., Nizet V. (2014). Subterfuge and sabotage: Evasion of host innate defenses by invasive gram-positive bacterial pathogens. Annu. Rev. Microbiol..

[19] Brouwer S., Barnett T.C., Rivera-Hernandez T., Rohde M., Walker M.J. (2016). *Streptococcus pyogenes* adhesion and colonization. FEBS Lett..

[20] Rivera-Hernandez T., Carnathan D.G., Jones S., Cork A.J., Davies M.R., Moyle P.M., Toth I., Batzloff M.R., McCarthy J., Nizet V. (2019). An experimental group A Streptococcus vaccine that reduces pharyngitis and tonsillitis in a nonhuman primate model. mBio.

[21] Huang E., Maldonado A.Q., Kjellman C., Jordan S.C. (2022). Imlifidase for the treatment of anti-HLA antibody-mediated processes inkidney transplantation. Am. J. Transplant..

[22] Sjogren J., Lood R., Nageli A. (2020). On enzymatic remodeling of IgG glycosylation; unique tools with broad applications. Glycobiology.

[23] Happonen L., Collin M. (2024). Immunomodulating Enzymes from *Streptococcus pyogenes*—In Pathogenesis, as Biotechnological Tools, and as Biological Drugs. Microorganisms.

[24] Proft T., Fraser J.D. (2007). Streptococcal superantigens. Chem. Immunol. Allergy.

[25] Stevens D.L., Bryant A.E., Yan S. (1994). Invasive group A streptococcal infection: New concepts in antibiotic treatment. Int. J. Antimicrob..

[26] Allen U., Moore D. (2010). Invasive group A streptococcal disease: Management and chemoprophylaxis. Can. J. Infect. Dis. Med. Microbiol..

[27] Eagle H. (1952). Experimental approach to the problem of treatment failure with penicillin. I. Group A streptococcal infection in mice. Am. J. Med..

[28] Stevens D.L., Gibbons A.E., Bergstrom R., Winn V. (1988). The Eagle effect revisited: Efficacy of clindamycin, erythromycin, and penicillin in the treatment of streptococcal myositis. J. Infect. Dis..

[29] Gajdács M., Ábrók M., Lázár A., Burián K. (2020). Beta-Haemolytic group A, C and G streptococcal infections in southern Hungary: A 10-year population-based retrospective survey (2008–2017) and a review of the literature. Infect. Drug Resist..

[30] Jayakumar J.S., Niyas V.K.M., Arjun R. (2022). Group A streptococcal bacteremia: Ten years’ experience from a tertiary Care Center in South India. Indian J. Crit. Care Med. Peer-Rev. Off. Publ. Indian Soc. Crit. Care Med..

[31] Villalón P., Bárcena M., Medina-Pascual M.J., Garrido N., Pino-Rosa S., Carrasco G., Valdezate S. (2023). National Surveillance of tetracycline, erythromycin, and clindamycin resistance in invasive *Streptococcus pyogenes*: A retrospective study of the situation in Spain, 2007–2020. Antibiotics.

[32] Databáze Výsledků Studie “RESPIRAČNÍ PATOGENY” [online]. Dostupný na. https://apps.szu.cz/rp/rezistence.php.

[33] Chochua S., Metcalf B., Li Z., Mathis S., Tran T., Rivers J., Fleming-Dutra K.E., Li Y., McGee L., Beall B. (2022). Invasive group A streptococcal penicillin binding protein 2× variants associated with reduced susceptibility to β-lactam antibiotics in the United States, 2015–2021. Antimicrob. Agents Chemother..

[34] Raveendran A.V., Kumar A., Gangadharan S. (2019). Biomarkers and newer laboratory investigations in the diagnosis of sepsis. J. R. Coll. Physicians Edinb..

[35] Candel F.J., Sá M.B., Belda S., Bou G., Del Pozo J.L., Estrada O., Ferrer R., González Del Castillo J., Julián-Jiménez A., Martín-Loeches I. (2018). Current aspects in sepsis approach. Turning things around. Rev. Española Quimioter..

[36] Meisner M. (2002). Pathobiochemistry and clinical use of procalcitonin. Clin. Chim. Acta.

[37] Aziz S.A., Nelwan E.J., Sukrisman L., Suhendro S. (2018). Higher cut-off serum procalcitonin level for sepsis diagnosis in metastatic solid tumor patients. BMC Res. Notes.

[38] Wacker C., Prkno A., Brunkhorst F.M., Schlattmann P. (2013). Procalcitonin as diagnostic marker for sepsis: A systematic review and meta-analysis. Lancet Infect. Dis..

[39] Ullberg M., Özenci V. (2020). Identification and antimicrobial susceptibility testing of Gram-positive and Gram-negative bacteria from positive blood cultures using the Accelerate Pheno™ system. Eur. J. Clin. Microbiol. Infect. Dis..

[40] O’Loughlin R.E., Roberson A., Cieslalc P.R., Lynfield R., Gershman K., Craig A., Albanese B.A., Farley M.M., Barrett N.L., Spina N.L. (2007). The epidemiology of invasive group A streptococcal infection and potential vaccine implications: United States, 2000–2004. Clin Infect Dis..

[41] Bonnet M., Lagier J.C., Raoult D., Khelaifia S. (2019). Bacterial culture through selective and non-selective conditions: The evolution of culture media in clinical microbiology. New Microbes New Infect..

[42] Bursle E., Robson J. (2016). Non-culture methods for detecting infection. Aust. Prescr..

[43] Singer M., Deutschman C.S., Seymour C.W., Shankar-Hari M., Annane D., Bauer M., Bellomo R., Bernard G.R., Chiche J.D., Coopersmith C.M. (2016). The Third International Consensus Definitions for Sepsis and Septic Shock (Sepsis-3). JAMA.

[44] Dellinger R.P., Rhodes A., Evans L., Alhazzani W., Beale R., Jaeschke R., Machado F.R., Masur H., Osborn T., Parker M.M. (2023). Surviving Sepsis Campaign Guidelines 2021. Crit. Care Med..

[45] Arora S., Singh P., Singh P.M., Trikha A. (2015). Procalcitonin levels in survivors and nonsurvivors of sepsis: Systematic review and meta-analysis. Shock.

[46] Wagner N.M., Van Aken C., Butschkau A., Bierhansl L., Kellner P., Schleusener V., Seggewiss J., Vollmar B., Nöldge-Schomburg G., Roesner J.P. (2017). Procalcitonin impairs endothelial cell function and viability. Anesth. Analg..

[47] Adamkova V., Adamkova V.G., Lahoda Brodska H. (2020). Procalcitonin: A tricky biomarker for an initial choice of appropriate atb therapy!. Crit. Care.

[48] Adamkova V., Lahoda Brodska H., Adamkova V.G., Zima T. (2020). Can gram-negative-like biomarker values in *Streptococcus pyogenes* sepsis negatively influence right choice of initial antibiotic therapy?. Epidemiol. Mikrobiol. Imunol..

[49] Valderrama J.A., Nizet V. (2018). Group A *Streptococcus* encounters with host macrophages. Future Microbiol..

[50] Vanderschueren S., Deeren D., Knockaert D.C., Bobbaers H., Bossuyt X., Peetermans W. (2006). Extremely elevated C-reactive protein. Eur. J. Intern. Med..

[51] Gómez N.F.P., Del Pilar Sanz Martín M., Chong M., Cruz N.D.Z., Hernández R.M., Molina I., Sanz I.G., Tejerina A.F., Rueda F.R. (2024). Usefulness of Procalcitonin Levels for Predicting the Microbiological Orientation in Patients with Sepsis. J. Pers. Med..

[52] Garcia-Alvarez M., Marik P., Bellomo R. (2014). Sepsis-associated hyperlactatemia. Crit. Care.

[53] Rueddel T., Daniel O., Poidinger B., Weiss M., Bach F., Dey K., Häberle H., Kaisers U., Rüddel H., Schädler D. (2015). Hyperlactatemia is an independent predictor of mortality and denotes distinct subtypes of severe sepsis and septic shock. J. Crit. Care.

[54] Hu J., Jin Q., Fang H., Zhang W. (2024). Evaluating the predictive value of initial lactate/albumin ratios in determining prognosis of sepsis patients. Medicine.

[55] Dou Q.L., Liu J., Zhang W., Wang C.W., Gu Y., Li N., Hu R., Hsu W.T., Huang A.H., Tong H.S. (2022). Dynamic changes in heparin-binding protein as a prognostic biomarker for 30-day mortality in sepsis patients in the intensive care unit. Sci. Rep..

[56] Svobodová E., Drábek T., Brodská H. (2022). Pervitin Intoxication with Two-peak Massive Myoglobinemia, Acute Kidney Injury and Marked Procalcitonin Increase Not Associated with Sepsis. Prague Med. Rep..

[57] Raju N.A., Rao S.V., Joel J.C., Jacob G.G., Anil A.K., Gowri S.M., Kandasamy S. (2017). Predictive Value of Serum Myoglobin and Creatine Phosphokinase for Development of Acute Kidney Injury in Traumatic Rhabdomyolysis. Indian J. Crit. Care Med..

[58] Uchino S., Kellum J.A., Bellomo R., Doig G.S., Morimatsu H., Morgera S., Schetz M., Tan I., Bouman C., Macedo E. (2005). Acute renal failure in critically ill patients: A multinational, multicenter study. JAMA.

[59] Bellomo R., Kellum J.A., Ronco C., Wald R., Martensson J., Maiden M., Bagshaw S.M., Glassford N.J., Lankadeva Y., Vaara S.T. (2017). Acute kidney injury in sepsis. Intensive Care Med..

[60] Hoste E.A., Bagshaw S.M., Bellomo R., Cely C.M., Colman R., Cruz D.N., Edipidis K., Forni L.G., Gomersall C.D., Govil D. (2015). Epidemiology of acute kidney injury in critically ill patients: The multinational AKI-EPI study. Intensive Care Med..

[61] Vincent J.L., Sakr Y., Sprung C.L., Ranieri V.M., Reinhart K., Gerlach H., Moreno R., Carlet J., Le Gall J.R., Payen D. (2006). Sepsis in European intensive care units: Results of the SOAP study. Crit. Care Med..

[62] Andreoni F., Zurcher C., Tarnutzer A., Schilcher K., Neff A., Keller N., Marques M.E., Poyart C., Schuepbach R.A., Zinkernagel A.S. (2017). Clindamycin affects group A streptococcus virulence factors and improves clinical outcome. J. Infect. Dis..

[63] Mulla Z.D., Leaverton P.E., Wiersma S.T. (2003). Invasive group A streptococcal infections in Florida. South Med. J..

[64] Linner A., Darenberg J., Sjolin J., Henriques-Normarlc B., Norrby-Teglund A. (2014). Clinical efficacy of polyspecific intravenous immunoglobulin therapy in patients with streptococcal toxic shock syndrome: A comparative observational study. Clin. Infect. Dis..

[65] Couture-Cossette A., Carignan A., Mercier A., Desruisseaux C., Valiquette L., Pépin J. (2018). Secular trends in incidence of invasive beta-hemolytic streptococci and efficacy of adjunctive therapy in Quebec, Canada, 1996–2016. PLoS ONE.

[66] Armengol Álvarez L., Van de Sijpe G., Desmet S., Metsemakers W.-J., Spriet I., Allegaert K., Rozenski J. (2022). Ways to Improve Insights into Clindamycin Pharmacology and Pharmacokinetics Tailored to Practice. Antibiotics.

[67] White B.P., Siegrist E.A. (2021). Increasing clindamycin resistance in group A Streptococcus. Lancet Infect. Dis..

[68] Bamford A., Whittaker E. (2023). Resurgence of group A streptococcal disease in children. BMJ.

[69] Bagcchi S. (2023). Surge of invasive group A streptococcus disease. Lancet Infect. Dis..

[70] Nash K., Lai J., Sandhu K., Chandan J.S., Shantikumar S., Ogunlayi F., Coleman P.C. (2022). Impact of national COVID-19 restrictions on incidence of notifiable communicable diseases in England: An interrupted time series analysis. BMC Public Health.

[71] Johannesen T.B., Munkstrup C., Edslev S.M., Baig S., Nielsen S., Funk T., Kristensen D.K., Jacobsen L.H., Ravn S.F., Bindslev N. (2023). Increase in invasive group A streptococcal infections and emergence of novel, rapidly expanding sub-lineage of the virulent *Streptococcus pyogenes* M1 clone, Denmark, 2023. Eurosurveillance.

[72] Zhi X., Li H.K., Li H., Loboda Z., Charles S., Vieira A., Huse K., Jauneikaite E., Reeves L., Mok K.Y. (2023). Emerging invasive group A streptococcus M1UK Lineage detected by allele-specific PCR, England, 2020. Emerg. Infect. Dis..

[73] Lynskey N.N., Jauneikaite E., Li H.K., Zhi X., Turner C.E., Mosavie M., Pearson M., Asai M., Lobkowicz L., Chow J.Y. (2019). Emergence of dominant toxigenic M1T1 *Streptococcus pyogenes* clone during increased scarlet fever activity in England: A population-based molecular epidemiological study. Lancet Infect. Dis.

[74] Alcolea-Medina A., Snell L.B., Alder C., Charalampous T., Williams T.G.S., Tan M.K.I., Al-Yaakoubi N., Humayun G., Newsholme W., Synnovis Microbiology Laboratory Group (2023). The ongoing *Streptococcus pyogenes* (Group A *Streptococcus*) outbreak in London, United Kingdom, in December 2022: A molecular epidemiology study. Clin. Microbiol. Infect..

[75] Guy R., Henderson K.L., Coelho J., Hughes H., Mason E.L., Gerver S.M., Demirjian A., Watson C., Sharp A., Brown C.S. (2023). Increase in invasive group A streptococcal infection notifications, England, 2022. Eurosurveillance.

[76] Apte R.S., Chen D.S., Ferrara N. (2019). VEGF in Signaling and Disease: Beyond Discovery and Development. Cell.

[77] Lu S.L., Kuo C.F., Chen H.W., Yang Y.S., Liu C.C., Anderson R., Wu J.J., Lin Y.S. (2015). Insufficient Acidification of Autophagosomes Facilitates Group A *Streptococcus* Survival and Growth in Endothelial Cells. mBio.

[78] Lu S.L., Kawabata T., Cheng Y.L., Omori H., Hamasaki M., Kusaba T., Iwamoto R., Arimoto H., Noda T., Lin Y.S. (2017). Endothelial cells are intrinsically defective in xenophagy of *Streptococcus pyogenes*. PLoS Pathog..

[79] Semeraro F., Morescalchi F., Duse S., Gambicorti E., Cancarini A., Costagliola C. (2015). Pharmacokinetic and Pharmacodynamic Properties of Anti-VEGF Drugs After Intravitreal Injection. Curr. Drug Metab..

[80] Touyz R.M., Herrmann S.M.S., Herrmann J. (2018). Vascular toxicities with VEGF inhibitor therapies-focus on hypertension and arterial thrombotic events. J. Am. Soc. Hypertens..

